# Aquaporin-9-expressing neutrophils are required for the establishment of contact hypersensitivity

**DOI:** 10.1038/srep15319

**Published:** 2015-10-22

**Authors:** Catharina Sagita Moniaga, Sachiko Watanabe, Tetsuya Honda, Søren Nielsen, Mariko Hara-Chikuma

**Affiliations:** 1 Center for Innovation in Immunoregulative Technology and Therapeutics; 2 Department of Dermatology, Graduate School of Medicine, Kyoto University, Kyoto 606-8501, Japan; 3 Department of Health Science and Technology, Aalborg University, Aalborg 9220, Denmark

## Abstract

Aquaporin-9 (AQP9), a water/glycerol channel protein, is expressed in several immune cells including neutrophils; however, its role in immune response remains unknown. Here we show the involvement of AQP9 in hapten-induced contact hypersensitivity (CHS), as a murine model of skin allergic contact dermatitis, using AQP9 knockout (AQP9^−/−^) mice. First, the CHS response to hapten dinitrofluorobenzene (DNFB) was impaired in AQP9^−/−^ mice compared with wild-type (WT) mice. Adoptive transfer of sensitized AQP9^−/−^ draining lymph node (dLN) cells into WT recipients resulted in a reduced CHS response, indicating impaired sensitization in AQP9^−/−^ mice. Second, administration of WT neutrophils into AQP9^−/−^ mice during sensitization rescued the impaired CHS response. Neutrophil recruitment to dLNs upon hapten application was attenuated by AQP9 deficiency. Coincidentally, AQP9^−/−^ neutrophils showed a reduced CC-chemokine receptor 7 (CCR7) ligand-induced migration efficacy, which was attributed to the attenuated recruitment of neutrophils to dLNs. Furthermore, we found that neutrophil deficiency, observed in AQP9^−/−^ or neutrophil-depleted mice, decreased IL-17A production by dLN cells, which might be responsible for T cell activation during a subsequent CHS response. Taken together, these findings suggest that AQP9 is required for the development of sensitization during cutaneous acquired immune responses via regulating neutrophil function.

Allergic contact dermatitis (ACD) is one of the most prevalent skin diseases consisting of sensitization and elicitation phases^1^,^2^. Advanced studies of hapten-induced contact hypersensitivity (CHS) as a murine model of ACD have expanded our understanding of the mechanism of allergic reactions occurring in the skin, especially the specific roles of a variety of immune cells. During the sensitization phase, hapten-bearing cutaneous dendritic cells (DCs) migrate into skin draining lymph nodes (dLNs), where the presentation of antigens to naïve T cells and subsequent T cell priming occur. During the elicitation phase, re-exposure to cognate hapten results in the recruitment of antigen-specific T cells to the site of allergen challenge and T cell-mediated tissue damage^2^. Increasing evidence shows that several subsets of immune cells, including various types of T cells (Th1, Th17, regulatory, and natural killer), DCs (dermal DCs and Langerhans cells), and mast cells, work synergistically in the skin and its dLNs to exert antigen presentation and T cell activation during the development of sensitization in CHS^3^,^4^,^5^,^6^,^7^.

Neutrophils have long been considered as the final effector cells of an acute inflammatory response. On the other hand, accumulating evidence has extended the function of neutrophils to include activation and regulation during innate and adaptive immune responses^8^,^9^. Several studies have focused on the recruitment of neutrophils into lymph nodes (LNs) in response to infection or immunization^10^,^11^,^12^, raising the possibility that neutrophils may modulate immune responses within LNs. With respect to the role of neutrophils in CHS, neutrophils were known to be important for the elicitation phase in which neutrophil recruitment to the hapten-challenged site led to the infiltration of hapten-specific CD8^+^ T cells and development of a CHS response^13^,^14^. In contrast, a more recent study suggested the requirement of neutrophils for both the sensitization and elicitation phase of CHS^15^. However, it remains unclear exactly how neutrophils exert its effect on the development of CHS.

Aquaporins (AQPs), which consist of 13 subsets in mammals (named AQP 0–12), are membrane channel proteins that participate in various biological functions, including cell proliferation and migration^16^,^17^,^18^. AQP9, our focus in this study, is expressed in a variety of cells such as erythrocytes, hepatocytes, adipocytes, and neutrophils^19^,^20^,^21^. Studies using AQP9 knockout (AQP9^−/−^) mice showed that AQP9, via its glycerol transport function, is involved in hepatic glycerol metabolism^22^ and in malarial infection^19^, whereas no phenotypic change in the skin or immune system under steady-state conditions has been reported. *In vitro* studies have suggested the involvement of AQP9 in cell motility and polarization; however, there is little direct evidence for this involvement^21^,^23^,^24^.

Here we tested the hypothesis that AQP9 has an important role in cutaneous immune responses, particularly via its expression in neutrophils. Our study using AQP9^−/−^ mice and a CHS murine model has shown that AQP9-expressing neutrophils is required for the sensitization phase of CHS through the cell migration function.

## Results

### Normal cellularity and subpopulations of immune cells in AQP9^−/−^ mice

**Figure 1:**
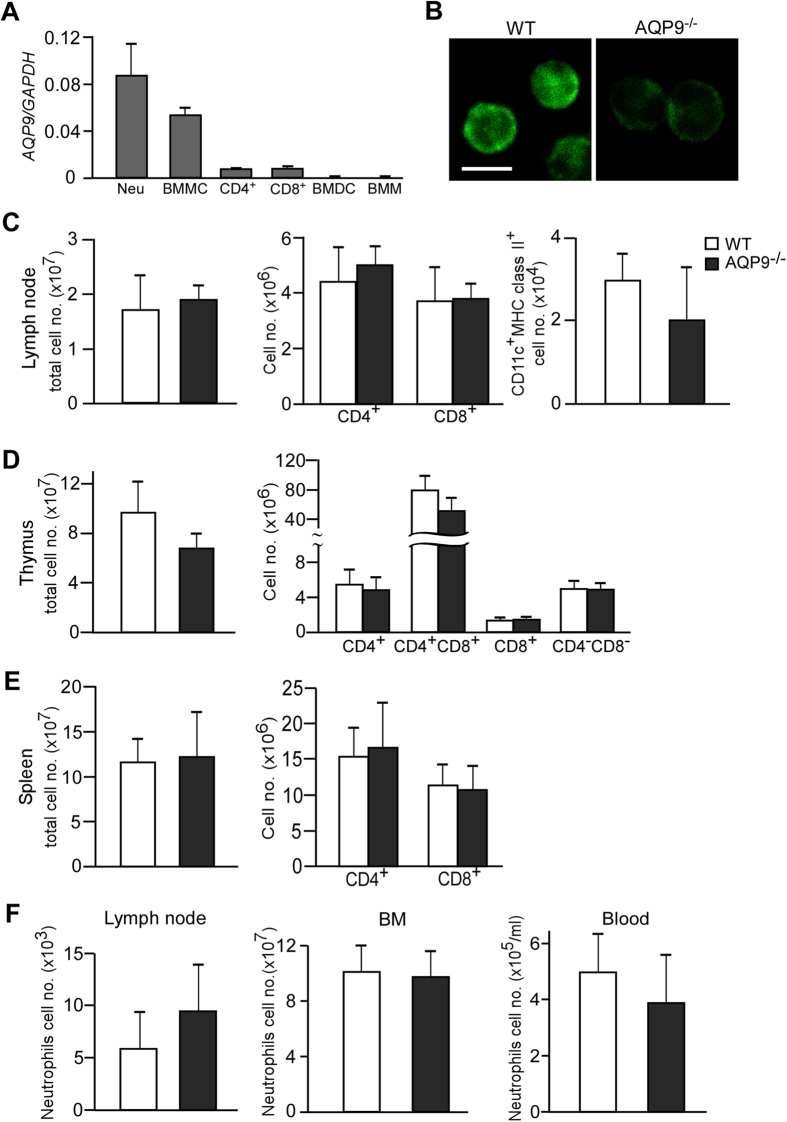
Normal cellularity and subpopulations of cells in AQP9^−/−^ mice. (**A**) The messenger RNA expression levels of *AQP9* in Macs-sorted neutrophils (Neu), BMMC, CD4^+^ and CD8^+^ T cells, BMDC, and BMM from C57BL/6 mice were assessed by real-time PCR. Data are expressed as the *AQP9/GAPDH* ratio. (**B**) Immunofluorescence with anti-AQP9 in neutrophils. Scale bars, 10 μm. (**C**–**E**) Cell population analysis in the skin dLNs (**C**), thymus (**D**), and spleen (**E**). The numbers of total cells, CD4^+^, CD8^+^ T cells, and CD11c^+^ MHC class II^+^ cells from WT and AQP9^−/−^ mice are shown (n = 3–5). (**F**) The neutrophil numbers in skin dLN, BM, and blood under steady state (n = 4). BMMC, Bone-marrow mast cell; BMDC, Bone-marrow dendritic cell; BMM, Bone-marrow macrophage; BM, Bone-marrow. All data are presented as the mean ± SD.

Previous studies have shown that AQP9 is expressed in a variety of cell types, including hepatocytes, epididymis, keratinocytes^22^, erythrocytes^19^, osteoclasts^25^, and adipocytes^20^ in wild-type (WT) mice. However, little is known about the expression of AQP9 in immune cells in mice. Quantitative real-time RT-PCR analysis showed high levels of *AQP9* expression in neutrophils, followed by bone marrow mast cells (BMMCs), whereas its expression levels in CD4^+^ and CD8^+^ T cells, bone marrow dendritic cells (BMDCs), and bone marrow macrophages (BMMs) were quite low (Fig. 1A). Immunofluorescence showed that AQP9 was localized in the cytosol and on the plasma membrane of WT neutrophils under steady-state conditions (Fig. 1B).

To determine the function of AQP9 in the immune system *in vivo*, phenotypic analysis was conducted on skin dLNs, spleen, and thymus from WT and AQP9^−/−^ mice. The CD4^+^ and CD8^+^ T cell counts in skin dLNs, thymus, and spleen were similar between WT and AQP9^−/−^ mice, as were CD11c^+^ MHC class II^+^ populations in skin dLNs (Fig. 1C–E). With regard to neutrophils, the cell numbers in skin dLNs, BM, and blood were also comparable between WT and AQP9^−/−^ mice (Fig. 1F). These data suggested that the germline AQP9 deficiency does not affect the homeostasis of the immune system under steady-state conditions.

### Suppression of CHS with impaired sensitization in AQP9^−/−^ mice

**Figure 2:**
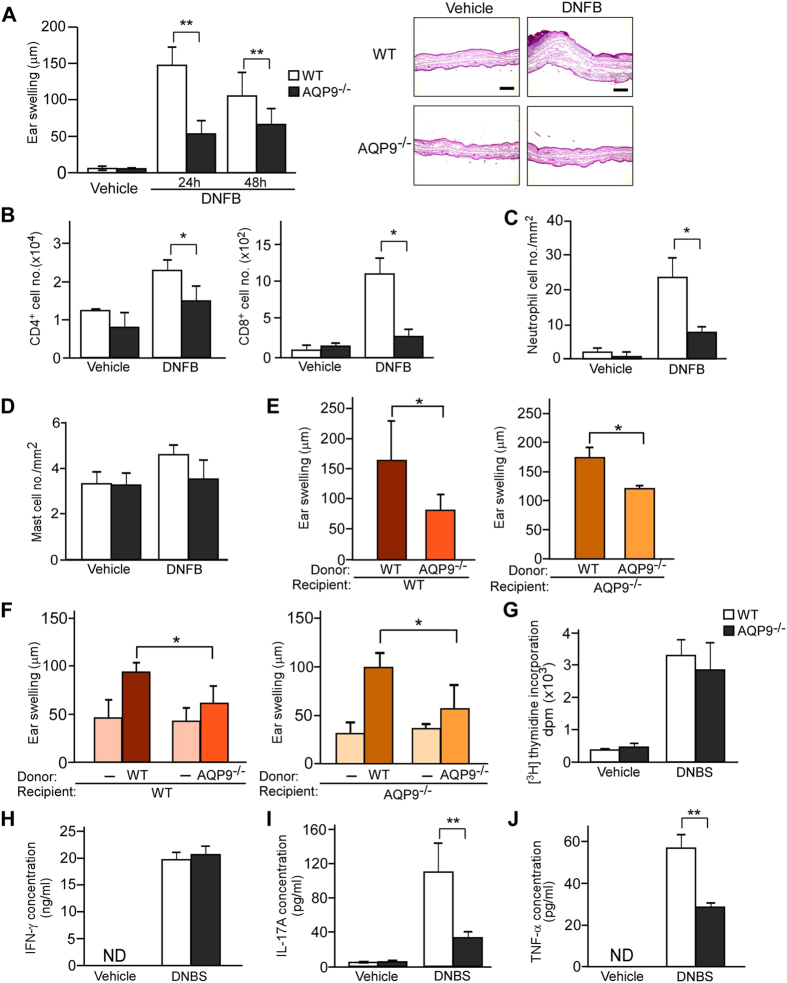
Suppression of CHS with impaired sensitization in AQP9^−/−^ mice. (**A**) Mice were sensitized with DNFB, and challenged 5 days later on the ear. (left) The ear swelling was measured at 24 and 48 h after challenge (n = 5, **p < 0.01). (right) HE staining of the ears of sensitized WT and AQP9^−/−^ at 24 h after challenge with DNFB. Scale bars, 200 μm. (**B–D**) Cell numbers in the ear skin at 24 h after challenge with DNFB. (**B**) CD4^+^ and CD8^+^ T cells were analyzed by FACS (n = 4, *p < 0.05). Neutrophils (**C**) and mast cells (**D**) were analyzed from Giemsa and Toluidin blue staining, respectively. Positive cells number was calculated on mm^2^ per skin area (n = 4, *p < 0.05). (**E**) CHS test using BM cell transferred mice. WT or AQP9^−/−^ mice received transplants of BM cells from WT and AQP9^−/−^ mice. The CHS test was performed with DNFB two months later. Ear swellings at 24 h after challenge are shown (n = 3–6; *p < 0.05). (**F**) Skin dLN cells from sensitized donor WT and AQP9^−/−^ mice were injected intradermally (2 × 10^5^ cells) into the ears of recipient WT (left) and AQP9^−/−^ (right) mice. Ear swellings at 24 h after challenge are shown (n = 4, *p < 0.05). (**G–J**) The skin dLN cells from sensitized WT and AQP9^−/−^ mice were collected at 5 days after DNFB application and cultured for 3 days with or without DNBS (100 μg/ml). (**G**) Cell proliferation was measured by [^3^H] thymidine incorporation. (**H–J**) The amount of IFN-γ, IL-17A and TNF-α in the culture medium were measured by ELISA (n = 5, **p < 0.01). ND = not detected. All data are presented as the mean ± SD.

To investigate the role of AQP9 in cutaneous acquired immune responses, we used a hapten-induced CHS murine model. Ear swelling, the hallmark of CHS, was decreased in AQP9^−/−^ mice as compared to WT mice at both 24 and 48 h after the challenge with hapten dinitrofluorobenzene (DNFB) (Fig. 2A, left). A single application of DNFB or phorbol myristate acetate (PMA) induced identical ear swellings between WT and AQP9^−/−^ mice, suggesting that an immune response is responsible for the differences in CHS responses (Supplementary Fig. S1A). Histology of the challenged skin showed less lymphocyte infiltration and epidermal hyperplasia in AQP9^−/−^ than WT skin (Fig. 2A, right). Consistent with these results, reduced numbers of infiltrating CD4^+^ and CD8^+^ T cells (Fig. 2B) and neutrophils (Fig. 2C; Supplementary Fig. S1B) were observed in the skin of AQP9^−/−^ mice after challenge compared to that of WT mice. Mast cell counts were comparable between WT and AQP9^−/−^ mice (Fig. 2D).

To investigate the potential significance of AQP9 expression in immune cells, lethally irradiated WT and AQP9^−/−^ mice (recipients) were reconstituted with bone marrow (BM) cells from WT and AQP9^−/−^ mice (donors) (Supplementary Fig. S2A). Ear swelling was impaired in the chimeric mice reconstituted with AQP9^−/−^ BM cells compared with the response in mice reconstituted with WT BM cells (Fig. 2E). These data indicate that the CHS response requires AQP9 expression on hematopoietic cells.

To investigate the sensitization phase of CHS in WT and AQP9^−/−^ mice, we performed a CHS test using subcutaneous adoptive transfer. Skin dLN cells from DNFB-sensitized WT and AQP9^−/−^ mice were injected subcutaneously into the ears of naïve recipient WT or AQP9^−/−^ mice, which were challenged immediately with DNFB. As shown in Fig. 2F, the mice injected with AQP9^−/−^ cells exhibited a significantly suppressed ear swelling as compared with WT cells transferred, indicating the impaired sensitization in AQP9^−/−^ mice. Flow cytometry (FACS) analysis of skin dLN cells at 5 days after DNFB sensitization showed that total immune cell number, CD4^+^ and CD8^+^ T cell counts were similar between WT and AQP9^−/−^ mice (Supplementary Fig. S2B–D). The increase in neutrophils number induced by DNFB sensitization was suppressed in AQP9^−/−^ compared to WT mice (Supplementary Fig. S2E).

To further dissect the role of AQP9 in sensitization during the CHS response, antigen-specific T cell proliferation and differentiation were determined *in vitro*. At 5 days after DNFB sensitization, the skin dLN cells from WT and AQP9^−/−^ mice were challenged with 2,4-dinitrobenzenesulfonic acid dehydrate (DNBS), a water-soluble compound with the same antigenicity as DNFB. In the presence of DNBS, the incorporation of [^3^H]-thymidine (Fig. 2G) and the levels of IFN-γ in the culture supernatant (Fig. 2H) were comparable between WT and AQP9^−/−^ cells. On the other hand, the DNBS-induced increases in IL-17A and TNF-α levels were markedly lower in dLN cells from AQP9^−/−^ mice as compared with those from WT mice (Fig. 2I,J).

Collectively, our results indicate that AQP9 is required for the development of sensitization in the CHS response. Based on the previous studies showing that IL-17 has a stimulatory role in the development of the CHS response^26^,^27^, we assume that suppressed production of IL-17A in LN cells might primarily contribute to the impaired CHS in AQP9^−/−^ mice.

### Involvement of neutrophils in CHS response and IL-17A production through AQP9 expression

**Figure 3:**
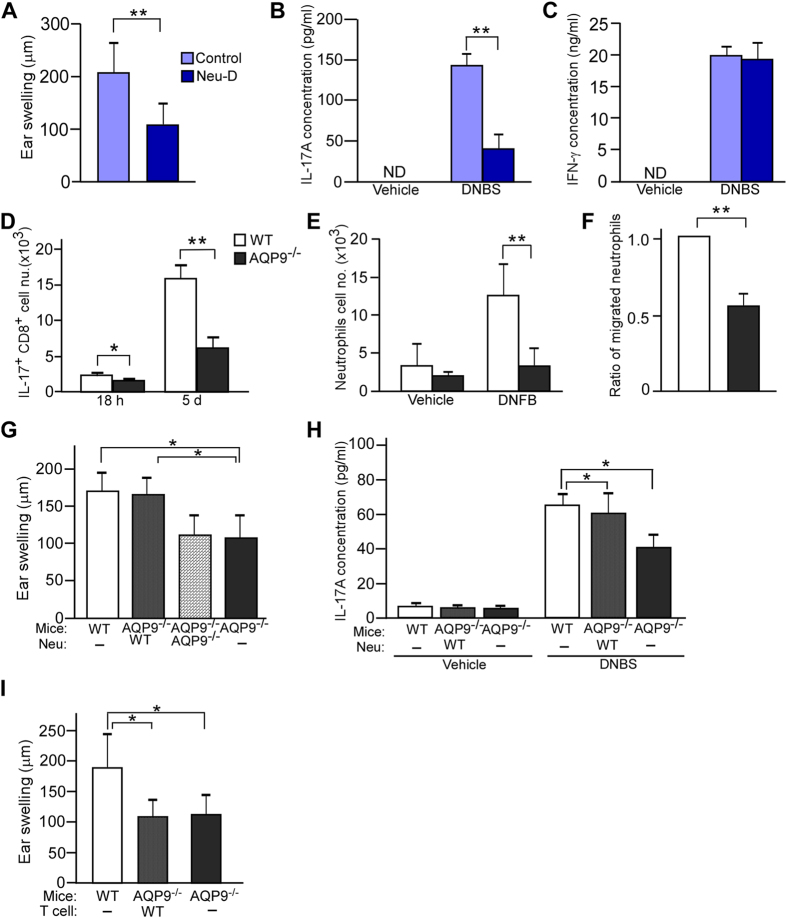
Involvement of neutrophils in CHS and IL-17A production through AQP9 expression. (**A–C**) WT mice were injected intraperitoneally with anti-Ly6G mAb (1 mg/head) at 1 day before and 2 days after sensitization. (**A**) Skin dLN cells from DNFB-sensitized control or anti-Ly6G antibody administered (Neu-D) mice were injected intradermally (2 × 10^5^ cells) into the ears of recipient naïve WT mice, and recipient mice were challenged with 0.5% DNFB. Ear swelling at 24 h after challenge is shown (n = 5; **p < 0.01). IL-17A (**B**) and IFN-γ (**C**) concentration in the culture medium of skin dLN cells were measured by ELISA (n = 5; **p < 0.01). ND = not detected. (**D**) Skin dLN cells from WT and AQP9^−/−^ mice were collected at 18 h and 5 days after DNFB sensitization for intracellular IL-17A staining by FACS analysis. Cells were cultured for 2 h with PMA (50 ng/ml) and ionomycin (1 μM) in the presence of GolgiStop. (**E**) Neutrophils number in skin dLNs was analyzed at 18 h after DNFB applications (n = 4; **p < 0.01). (**F**) BM-neutrophils of DNFB-applied WT and AQP9^−/−^ mice were labeled with CMFDA, and injected intravenously to WT recipient mice. The labeled neutrophils in dLNs were counted at 18 h after sensitization in recipient mice. Data are expressed as the ratio of recruited neutrophils (n = 3; **p < 0.01). (**G–H**) Neutrophils were isolated from BM cells at 18 h after DNFB application. WT or AQP9^−/−^ neutrophils were transferred to AQP9^−/−^ mice before and 1 day after sensitization with DNFB. Ear swelling at 24 h after challenge was measured (**G**) (n = 4; *p < 0.05). The concentration of IL-17A in the culture medium of skin dLN cells were measured by ELISA (H) (n = 4–5; *p < 0.05). (**I**) Sorted-T cells were collected from WT mice and transferred intravenously to AQP9^−/−^ mice before and 1 day after DNFB sensitization. Ear swelling at 24 h after challenge was measured (n = 4–6, *p < 0.05). All data are presented as the mean ± SD.

We next tested the assumption that impaired CHS in AQP9^−/−^ mice was attributed to suppressed production of IL-17A through neutrophil function. First, we examined whether neutrophils were required for IL-17A production and the development of sensitization during the CHS response. Neutrophils were depleted in donor WT mice by using anti-mouse Ly6G Ab (clone 1A8) during the sensitization phase and neutrophil depletion was confirmed by FACS at 5 days after sensitization (Supplementary Fig. S3A). We also verified that the administration of anti-Ly6G Ab did not affect the T and B cell populations (Supplementary Fig. S3B, C). When we injected sensitized dLN cells from the neutrophil-depleted mice subcutaneously into the ears of naïve recipient WT mice and then applied DNFB, the CHS response was significantly suppressed compared to the mice injected with sensitized cells from control mice (Fig. 3A). In this context, the IL-17A level produced from skin dLN cells with DNBS re-stimulation *in vitro* was remarkably attenuated by neutrophil depletion (Fig. 3B), whereas the IFN-γ level was unaffected (Fig. 3C).

A previous study demonstrated that IL-17 was primarily produced by CD8^+^ T cells during CHS^28^. In contrast, a recent study showed that neutrophils can produce IL-17, such as in the mouse kidney ischemia-reperfusion injury^29^ or in *in vivo* models of skin inflammation with histological features of human psoriasis^30^. In agreement with the previous study^28^, we found that CD8^+^ T cells, but not neutrophils, mainly produced IL-17A in skin dLNs at 18 h and 5 days post-sensitization with DNFB (supplementary Fig. S4A). In this setting, the numbers of IL17^+^ CD8^+^ T cells were significantly greater in WT than in AQP9^−/−^ mice after DNFB application (Fig. 3D).

We next assessed neutrophil distribution after DNFB sensitization. We found that the percentage of neutrophils in skin dLNs was remarkably increased at an early time point after DNFB application, peaking at 18 h (Supplementary Fig. S4B). AQP9^−/−^ mice showed markedly fewer numbers of neutrophils in skin dLNs at 18 h after sensitization compared to WT mice (Fig. 3E), whereas the number of neutrophils in BM and blood were similar (Supplementary Fig. S4C). These results suggested that AQP9 deficiency decreased the accumulation of neutrophils in skin dLNs during the sensitization phase of CHS.

An essential step in the sensitization phase for CHS is the migration of hapten-bearing cutaneous DCs into the skin dLNs. We verified that the number of CD11c^+^ MHC class II^+^ FITC^+^ DCs that migrated from the skin to the dLNs after FITC application were comparable between WT and AQP9^−/−^ mice (Supplementary Fig. S5).

We next determined the involvement of AQP9 in neutrophil recruitment to skin dLNs *in vivo*. We adoptively transferred sensitized WT or AQP9^−/−^ neutrophils, labeled with a cell-tracking dye (CMFDA), into naïve WT mice. The number of CMFDA^+^-neutrophils accumulated in skin dLNs were significantly lower in AQP9^−/−^ cells compared with that of WT cells (Fig. 3F), indicating that neutrophil recruitment to dLNs after sensitization was modulated by AQP9. To further confirm the involvement of neutrophils in CHS and IL-17A production, we examined whether the reconstitution of WT neutrophils into AQP9^−/−^ mice could restore the impaired CHS response and IL-17A production. As shown in Fig. 3G,H, transferred of sensitized WT neutrophils to AQP9^−/−^ mice during sensitization could restore the CHS response and IL-17A production of dLN cells, suggesting a critical role for neutrophils in the sensitization phase of CHS. In contrast, the impaired CHS in AQP9^−/−^ mice was not ameliorated by WT T cells transfer during sensitization (Fig. 3I). Taken together, our findings support an essential role for neutrophils mediated by AQP9 function in the development of sensitization during CHS.

### Increased expressions of costimulatory molecules in neutrophils during CHS

**Figure 4:**
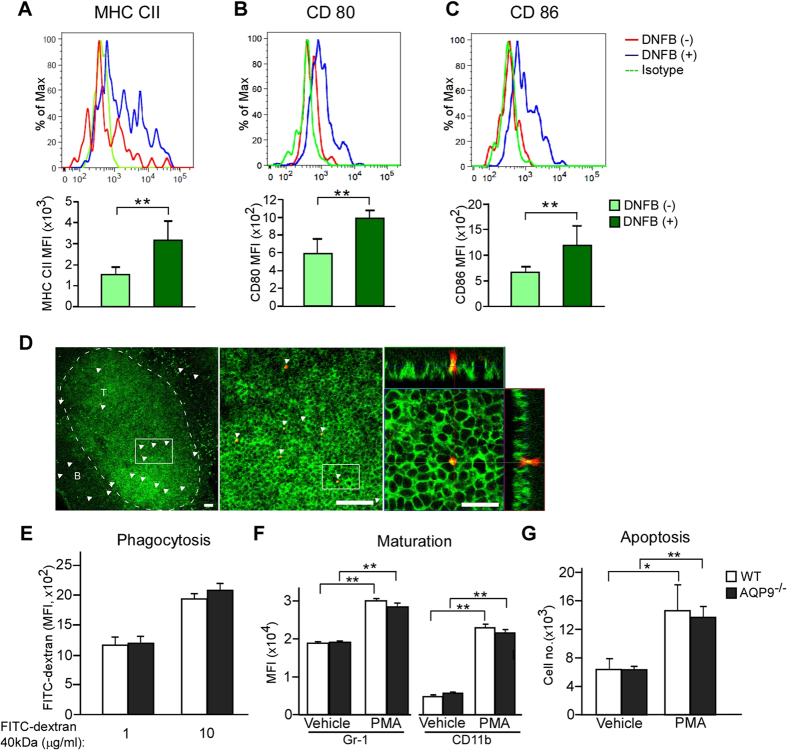
Increased expressions of costimulatory molecules in neutrophils during CHS. (**A–C**) Single-cell suspensions were collected from skin dLNs at 18 h after DNFB application. Neutrophil was defined as CD11b^+^ Gr-1^+^ F4/80^−^ cells by FACS analysis. (upper) The expression of MHC class II (**A**), CD80 (**B**), and CD86 (**C**) on neutrophils from DNFB applied or non-applied WT mice. (lower) The expression levels as mean fluorescence intensity (MFI) (n = 5; **p < 0.01). (**D**) Representative confocal image of immunofluorescence in WT skin dLNs at 24 h after DNFB application stained with anti-Gr-1 (for neutrophils, red) and anti-CD8 (for CD8^+^ T cells, green). Right panel shows focal planes that are parallel and perpendicular to the epithelium. Arrowheads indicate neutrophils. Scale bars = 100 μm (left, middle) and 50 μm (right). (**E**) BM cells from WT or AQP9^−/−^ mice were incubated with FITC-dextran (40 kDa, 1 or 10 μg/ml) for 45 min at 37 °C. MFI of internalized FITC in neutrophils was analyzed by FACS (n = 3). (**F**,**G**) BM cells from WT or AQP9^−/−^ mice were incubated with or without PMA (10 ng/ml) for 10 min (maturation) or 24 h (apoptosis) at 37 °C. (**F**) The maturation status was defined as the MFI of Gr-1^+^ and CD11b^+^ gated on neutrophils (n = 3; **p < 0.01). (**G**) The apoptotic cells were defined as CD11b^+^ Gr-1^+^ Annexin V^+^ 7AAD^+^ cells by FACS analysis (n = 4; *p<0.05; **p < 0.01). All data are presented as the mean ± SD.

Neutrophils have been reported to acquire antigen-presenting cell (APC) type functions in the presence of peptide antigen OVA or within the inflamed bowel, in which they acquired up-regulated expression of MHC class II and costimulatory molecules^31^,^32^. Therefore we analyzed those surface molecules of neutrophils in dLNs during CHS. We found that the expression levels of MHC class II and costimulatory molecules CD80 and CD86 were upregulated at 18 h after DNFB sensitization (Fig. 4A–C). In this setting, neutrophils were localized in the T-cell zone of the dLNs and appeared to make contact with CD8^+^ T cells after DNFB sensitization (Fig. 4D).

We next sought to determine the general functions of neutrophil between WT and AQP9^−/−^ mice. We found that the phagocytosis ability against FITC-labeled dextran, which commonly used model substance for phagocytosis, was comparable between WT and AQP9^−/−^ cells (Fig. 4E). In addition, the maturation and apoptosis induced by PMA were also similar between WT and AQP9^−/−^ neutrophils (Fig. 4F,G).

Collectively, our data indicate that the DNFB-sensitized neutrophils acquired higher expression of MHC class II and costimulatory molecules CD80 and CD86, and were localized adjacent to CD8^+^ T cells in skin dLN, implying possible involvement of neutrophils in the development of the sensitization phase of CHS.

### Impaired migration and water transport induced by chemokines in AQP9^−/−^ neutrophils

**Figure 5:**
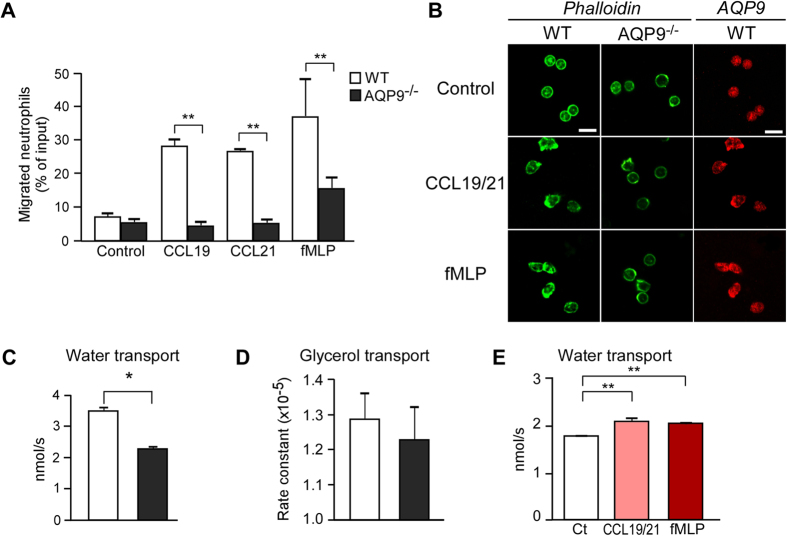
Impaired chemokine-induced migration and water permeability in AQP9-deficient neutrophils. (**A**) Chemotaxis assay of neutrophils toward CCL19 (200 ng/ml), CCL21 (200 ng/ml), or fMLP (100 nM) for 1 h (n = 5; **p < 0.01). Neutrophils were isolated from BM cells of DNFB-sensitized mice at 18 h. (**B**) Neutrophils of WT and AQP9^−/−^ mice were stimulated for 10 min with fMLP (100 nM) or CCL19/21 (200 ng/ml), and stained with phalloidin-AlexaFlour 488 for visualizing F-actin or with anti-AQP9 (cy3, red). Representative immunofluorescence microscopy. Bars, 20 μm. (**C**) The osmotic water permeability of neutrophils isolated from WT and AQP9^−/−^ mice was measured based on the time course of scattered light intensity in response to a 150-mM inwardly directed mannitol gradient generated by stopped flow at 22 °C. Averaged osmotic permeability coefficients (*Pf*; n = 5; *p < 0.05). (**D**) Glycerol permeability was measured in response to a 150-mM inwardly glycerol gradient by stopped flow at 30 °C (n = 5). (**E**) The osmotic water permeability of neutrophils from sensitized WT mice with CCL19/21 (200 ng/ml) or fMLP (100 nM) stimulations (*Pf*; n = 5; **p < 0.01). Ct = none treated cells. All data are presented as the mean ± SD.

It has been reported that the recruitment of neutrophils to LN is dependent on mainly CC-chemokine receptor 7 (CCR7)^12^. Therefore we examined neutrophil migration toward CCR7 ligands, e.g., CCL19 and CCL21, and fMLP, the most commonly used chemoattractant for neutrophils, by using a transwell chamber. We found that the efficiency of sensitized neutrophil migration toward CCL19, CCL21, or f MLP was markedly impaired in AQP9^−/−^ cells compared with WT cells (Fig. 5A). We verified comparable expression of the CCR7 receptor on neutrophils in skin dLNs of naïve and sensitized WT and AQP9^−/−^ mice (Supplementary Fig. S6). Once a chemoattractant binds to its cell surface receptor in neutrophils, a series of cytoplasmic events is triggered, which results in the reorganization of actin cytoskeleton, i.e., polymerization of monomeric G-actin to F-actin, which drives cell migration^33^,^34^. Consistent with previous findings, chemokine-stimulated WT neutrophils developed a polarized morphology at the leading edge, which was visualized for F-actin, whereas AQP9^−/−^ neutrophils failed to develop distinct uropods (Fig. 5B, left). Immunofluorescence staining showed that AQP9 was abundantly localized around the leading edge in chemokine-treated cells (Fig. 5B, right).

To further investigate the mechanism underlying AQP9-mediated neutrophils chemotaxis, we examined whether the membrane transport of water and glycerol, which may affect cellular activities, occurred in neutrophils in an AQP9-dependent manner. The osmotic water permeability of neutrophils was measured using the kinetics of scattered light intensity in response to osmotic challenge, as previously described^35^. Water transport capacity in response to a 150-mM inwardly directed mannitol gradient was significantly greater in WT than in AQP9^−/−^ neutrophils (Fig. 5C). In contrast, no significant difference was found in the glycerol transport capacity between the WT and AQP9^−/−^ neutrophils by stop-flow analysis (Fig. 5D).

Previous studies have shown that AQP-mediated water transport is implicated in cell migration^16^. To determine the involvement of AQP9-mediated water transport in chemotaxis, we measured the osmotic water permeability of sensitized WT neutrophils with CCL19/21 or fMLP stimulation. We found that both stimulants significantly increased water transport in WT (Fig. 5E) but not in AQP9^−/−^ neutrophils (data not shown).

Collectively, our findings support that AQP9 is involved in water transport in neutrophils during chemotaxis, which may have a prominent role in neutrophil migration.

## Discussion

Here we demonstrated that AQP9 is required for the establishment of CHS. First, AQP9^−/−^ mice showed a remarkably impaired CHS response to hapten DNFB. The adoptive transfer of AQP9^−/−^ dLN cells with DNFB application to the site of the secondary challenge impaired the CHS response, indicating that the development of sensitization was impaired in AQP9^−/−^ mice. Second, administration of WT neutrophils into AQP9^−/−^ mice during sensitization rescued the impaired CHS response. More specifically, neutrophil recruitment to dLNs upon hapten application was attenuated by AQP9 deficiency, suggesting the involvement of neutrophils within dLNs in a CHS response. Third, AQP9^−/−^ neutrophils showed reduced CCR7 ligand-induced migration efficacy, which was attributed to the attenuated recruitment of neutrophils to dLNs. Collectively, these results support the assertion that AQP9-expressing neutrophils are essential for the development of sensitization during cutaneous acquired immune responses via their migratory function.

Neutrophils are produced in the BM, circulate in the blood, and are rapidly recruited to sites of infection or dLNs in response to a variety of chemoattractants^12^,^36^. The results of previous studies have suggested that neutrophils recruited to dLNs can participate in the regulation of adaptive immune responses, either by acting directly via acquiring APC-like properties of presenting antigens and priming T cells^31^,^37^,^38^ or indirectly via APCs by mechanisms such as those that modulate DC maturation^39^,^40^. In the current study, our results revealed the indispensable role of neutrophils in the sensitization phase of CHS. After hapten application, neutrophils were recruited to skin dLNs with higher expression levels of the costimulatory molecules CD80 and CD86 and MHC class II molecules. An immunofluorescence assay revealed that neutrophils were co-localized and appeared to interact with CD8^+^ T cells in dLNs upon DNFB application. Although our results produced insufficient evidence regarding the ability of neutrophils to directly act as APCs on T cell activation after DNFB sensitization, our data implied that a substantial number of neutrophils recruited to skin dLNs may activate T cells via APC-like properties during the sensitization phase of CHS. Further studies are required to establish the precise mechanism(s), involving neutrophils, by which CD8^+^ T cell are activated during a CHS response.

In addition, the results of this study also demonstrated that neutrophil deficiency, observed in the AQP9^−/−^ or neutrophil-depleted mice, remarkably suppressed IL-17A production by dLN cells and particularly by CD8^+^ T cells. A previous study showed that cutaneous DNFB sensitization induced a population of hapten-specific IL-17-producing CD8^+^ T cells, which was required for an optimal CHS response^41^. Concurrently, IL-17-deficiency^26^ or IL-17 neutralization^27^ suppressed both the production of inflammatory cytokines in hapten-challenged skin tissues and CHS response. Based on these findings, we propose that the suppression of IL-17A production is responsible for the impairment of the CHS response in neutrophil-deficient conditions.

In agreement with our findings, novel results of a recent study established the requirement of neutrophils for the development of sensitization during CHS^15^. The study also revealed that hapten 2,4,6-trinitrochlorobenzene-induced sensitization subsequent to the CHS response was impaired in antibody (NIMP-R14)-mediated neutrophil-depleted mice due to suppression of IFN-γ production (derived from skin dLN cells) and DC migration to the dLN^15^. However, our findings indicate that DNFB-induced CHS in the neutrophil depletion caused by the 1A8 antibody did not abrogate IFN-γ production. Currently, there are several antibodies considered useful for neutrophil depletion; however, none of them depleted neutrophils completely. The antibodies NIMP-R14 and RB6-8C5 have a high degree of specificity for murine Ly-6G and Ly-6C, and consequently depleted not only neutrophils but also monocytes and macrophages^42^,^43^,^44^. On the other hand, the 1A8 antibody recognizes only Ly6G, which results in an adequate and more specific depletion of neutrophils, but less efficient than antibodies used in previous experiments^43^,^44^. Therefore, differences in the specific hapten and/or neutrophil antibodies used in a particular experiment may yield different populations of depleted cells, leading to disparate results including IFN-γ production.

A growing number of studies have shown the involvement of AQPs in cell migration; however, controversy remains regarding their specific molecular role^16^,^45^. The lack of AQP1, 3, or 4 has been observed in the cell-impaired migration of endothelial and epithelial cells, in which an AQP-mediated water flux was attributed to the facilitation of lamellipodial extension and cell migration with actin polymerization^45^. A recent update also showed that cancer cells in confined microenvironments utilized directed water permeation mediated by AQP5 to regulate cell volume through the flux of water, leading to net cell displacement and migration^46^. In contrast, our results demonstrated that AQP3 controlled the chemotaxis of T cells via the AQP3-mediated hydrogen peroxide (H_2_O_2_) transport function and its associated downstream cell signaling^47^. In the current study, we found that cell migration in response to CCR7 ligand chemokines was impaired in AQP9^−/−^ neutrophils. In this context, the same stimulation increased water transport, with a dramatic change in cell morphology in WT neutrophils, but not in AQP9^−/−^ cells. These results suggest that AQP9 is involved in cell volume changes and migration in response to chemokines, possibly via its water transport function. Previous studies showed that AQP9 can transport not only water but also a wide range of non-charged solutes including glycerol, purines, lactate, and arsenide^23^,^24^, all of which may change the metabolic pathway or cation conductance of the cells^48^,^49^. Therefore, we could not exclude the possibility that AQP9 may also have the ability to regulate cell migration via its transport function of other small solutes, such as lactate or H_2_O_2_. Further studies are warranted to identify the underlying mechanism of AQP9-involved cell migration.

In summary, our data highlight previously unknown roles for AQP9 and neutrophils in CHS. Furthermore, our findings support that AQP9 is a novel target for neutrophil-mediated diseases or undesired immune responses, such as ACD and psoriasis.

## Materials and Methods

### Mice

AQP9^−/−^ mice (C57BL/6 genetic background) were generated by targeted gene disruption as described previously^22^. All comparisons were performed between littermates. All animal experiments were carried out in accordance with the guidelines approved by the Committee on Animal Research of Kyoto University.

### Bone marrow neutrophil purification

Single cell suspensions of BM cells were collected from femur and tibia, and resuspended in RPMI supplemented with 2% heat-inactivated fetal calf serum (FCS). Cells were centrifuged and subjected to hypotonic red blood cell lysis. The cell suspension was layered on top of a step gradient consisting of 52, 69, and 78% Percoll diluted in PBS, and centrifuged at 500g for 30 min at 4 °C. Neutrophils were recovered at the interface of the 69 and 78% Percoll layers, as described previously^40^ with slightly modifications. More than 85% of isolated cells were mature neutrophils as assessed by FACS.

### CHS and adoptive transfer experiments

Mice were sensitized with 50 μl of 0.5% DNFB in acetone/olive oil (4:1) on abdomen skin. On day 5, the ears were challenged by the application of 20 μl of 0.3% DNFB. Ear swelling was measured with a micrometer after 24 and 48 h.

For adoptive transfer, cell suspensions obtained from the skin dLNs of 5 d DNFB-sensitized mice were injected subcutaneously (2 × 10^5^ per 30 μl PBS) into the ears of naïve WT or AQP9^−/−^ mice. The ears were immediately challenged by applying 20 μl of 0.5% DNFB to both sides of the ear. Ear thickness was measured after 24 h.

To track the transferred cells, BM cells were prepared from DNFB-sensitized mice. The isolated BM-neutrophils were stained with CMFDA for 20 min, washed, and injected intravenously (2 × 10^7^ cells/head). The regional LNs of recipient mice were excised at 18 h after DNFB application. Neutrophils and CMFDA^+^ cells were analyzed by FACS analysis.

For the reconstitution assay, BM-neutrophils from WT mice 18 h after DNFB sensitization were collected and transferred intravenously (1 × 10^7^ cells/head) into AQP9^−/−^ mice before and 1 day after sensitization.

For the T cell reconstitution assay, sorted T cells from spleen and skin dLNs of WT mice were collected and transferred intravenously (1 × 10^7^ cells/head) into AQP9^−/−^ mice before and 1 day after sensitization.

### Cell culture, reagents, antibodies, flow cytometry, and intracellular staining

Complete RPMI (cRPMI) culture medium consisting of RPMI 1640 (Invitrogen, Carlsbad, CA, USA) containing 10% heat-inactivated FCS, 5 × 10^−5^ M 2-mercaptoethanol, 2mM L-glutamine, 25 mM N-2-hydroxyethylpiperazine-N′-2-ethanesulfonic acid, 1 mM nonessential amino acids, 1 mM sodium pyruvate, 100 units/mL penicillin, and 100 μg/mL streptomycin, was used, unless otherwise indicated.

We purchased 2,4-dinitrofluorobenzen (DNFB) from Wako (Osaka, Japan) and 2,4-dinitrobenzenesulfonic acid dehydrate (DNBS) from MP Biomedicals, Inc. (CA, USA). Single cell suspensions were stained with monoclonal antibodies against CD4 (clone RM4-5), CD8 (clone 53-6.7), Gr-1 (clone RB6-8C5), CD11b (clone M1/70), F4/80 (clone BM8), IL-17A (clone 17B7), CCR7 (clone 4B12), CD80 (clone 16-10A1), CD86 (clone GL1), MHC Class II (clone M5/114.15.2) (eBioscience, San Diego, CA, USA), CD11c (clone N418), CD45 (clone 30-F11) (BioLegend, San Diego, CA, USA). The samples were analyzed using the flow cytometry FACS Canto II system (BD Biosciences) and FlowJo software (TreeStar, Ashland, OR, USA).

For whole skin cell suspensions, ear skin sheets were cut into small pieces with scissors in cRPMI medium and incubated for 25 min at 37 °C and 5% CO_2_ in type II collagenase (Sigma, St Louis, MO). For intracellular cytokine staining, cells were stimulated for 2 h with PMA (50 ng/ml) and ionomycin (1 μM) in the presence of GolgiStop^TM^ (BD Biosciences). After washing, cells were fixed and permeabilized with cytofix/cytoperm buffer (BD Biosciences), and stained with IL-17A.

### Bone marrow transplantation

For BM transplantation, red blood cells from WT and AQP9^-/-^ mice were subjected to hypotonic cell lysis. WT and AQP9^-/-^ recipients (8–10 weeks old) were γ-irradiated with two doses of 600 rad, 3 h apart. After irradiation, the mice received 10^6^ BM cells intravenously. This protocol consistently gave >90% reconstitution of the recipient by donor haematopoietic cells, as evaluated by separate transplantation experiments using BM from C57BL/6-CD45.1 congenic mice (Supplementary Fig.S2A). The CHS model was performed 2 months later.

### Neutrophils phagocytosis, maturation, and apoptosis assay

BM cells were collected from WT and AQP9^−/−^ mice and red blood cells were subjected to hypotonic cell lysis. For phagocytosis assay, FITC-Dextran (molecular mass 40kDa, 1 and 10 μg/ml; Sigma) was incubated with the cell solutions for 45 min in cRPMI (37 °C, 5% CO_2_). The cells were washed three times with cold PBS. The mean fluorescence intensity (MFI) of internalized FITC in CD11b^+^ Gr-1^+^ cells was analyzed by FACS.

For maturation or apoptosis assay, BM cells were incubated with or without PMA (10 ng/ml, Sigma) for 10 min or 24 h at 37 °C. The cells were washed two times with cold PBS. Flow cytometry was used to analyze MFI of Gr-1 and CD11b gated on neutrophils to observe the maturation status. The apoptotic neutrophils were defined as CD11b^+^ Gr-1^+^ Annexin V^+^ 7AAD^+^ cells by FACS analysis.

### Lymphocyte proliferation assay and cytokine production

For DNBS-dependent proliferation, single cell suspensions were prepared from skin dLNs of mice 5 days post-sensitization with DNFB. The skin dLN cells (1 × 10^6^) were cultured with or without DNBS (100 μg/ml) for 72 h, pulsed with 0.5 μCi [^3^H] thymidine for the last 24 h, and subjected to liquid scintillation counting. For the measurement of cytokine production, the culture supernatants were collected 72 h post-incubation. The amounts of IFN-γ, IL-17A, and TNF-α were measured by enzyme-linked immunosorbent assay (ELISA; eBiosciences).

### Antibody treatment

For depletion of neutrophils, mice were injected intraperitoneally (i.p) with rat anti-mouse Ly6G mAb, clone 1A8 (1 mg/200 μl/head, BioXcell, West Lebanon, NH) at 1 day before and 2 day after of sensitization. Neutrophils depletion was confirmed by FACS at 5 days after administration.

### Quantitative RT-PCR

mRNA was isolated using TRIzol (Invitrogen), according to the manufacturer’s instructions. Quantitative RT-PCR was performed as described previously, using the housekeeping gene glyceraldehyde-3-phosphate dehydrogenase (*GAPDH*) as a control^50^.

### Histochemistry and immunofluorescence

For the histological portion of the study, paraffin-embedded sections were stained with hematoxylin and eosin. Toluidine blue or Giemsa staining was used to detect mast cells or neutrophils, respectively. The stained cells were counted in 5 different areas, and average positive numbers were calculated on mm^2^ per skin area.

For cell polarization and AQP9 expression, cells were cultured on glass coverslips and stimulated with fMLP (Sigma), CCL19 (R&D, MN, USA), or CCL21 (R&D, MN, USA). Cells were fixed with 4% formalin in PBS, permeabilized with 0.1% saponin, and stained with phalloidine-Alexa488 (Invitrogen), and AQP9 (Alpha diagnostic international, San Antonio, Texas, USA), anti-rabbit secondary antibody (FITC, Sigma). Frozen LN sections were stained with anti-Ly6G/Alexa594 (clone 1A8, BioLegend) and anti-CD8/FITC (clone 16-10A1, eBioscience).

### Chemotaxis

BM-neutrophils from WT and AQP9^−/−^ mice 18 h after DNFB sensitization were deposited on the upper chamber containing a polycarbonate transwell membrane filter (5-μm pore size; Corning) for one hour. The lower chamber contained f MLP (100 nM), CCL19 (200 ng/ml), or CCL21 (200 ng/ml) in 0.5% BSA/RPMI medium. The recovered cells were analyzed with FACS analysis.

### Water and glycerol permeability assay

Water and glycerol permeability were measured using an SX20 stopped-flow spectrometer (Applied Photophysics). The BM-neutrophils were subjected to a 150-mM inwardly directed mannitol or glycerol gradient. The kinetics of the decreasing cell volumes was measured from the time course of 90 scattered light intensity at 450 nm wavelength. Osmotic water/glycerol permeability coefficients (*Pf*) were calculated as described previously^35^.

### Statistical Analysis

Unless otherwise indicated, data were presented as the means ± SD. *p* values were calculated according to the two-tailed Student’s *t*-test.

## Additional Information

**How to cite this article**: Moniaga, C. S. *et al.* Aquaporin-9-expressing neutrophils are required for the establishment of contact hypersensitivity. *Sci. Rep.* **5**, 15319; doi: 10.1038/srep15319 (2015).

## Supplementary Material

Supplementary Figures
