# Supplementary material for: Aquaporin-9-expressing neutrophils are required for the establishment of contact hypersensitivity
Source: Sci Rep. 2015 Oct 22;5:15319. doi: 10.1038/srep15319 (PMC4614820; doi:10.1038/srep15319)
Supplement: Supplementary Figures [file srep15319-s1.pdf]

Supplemental Material

Aquaporin-9-expressing neutrophils are required for the establishment of contact hypersensitivity

Catharina Sagita Moniaga[1], Sachiko Watanabe[1], Tetsuya Honda[1, 2], Søren Nielsen[3], Mariko Hara-Chikuma[1*]

[1]Center for Innovation in Immunoregulative Technology and Therapeutics, [2]Department of Dermatology, Graduate School of Medicine, Kyoto University, Kyoto 606-8501, Japan, [3]Department of Health Science and Technology, Aalborg University, Aalborg 9220, Denmark

Address correspondence to:
Mariko Hara-Chikuma, Ph.D.
Center for Innovation in Immunoregulative Technology and Therapeutics
Graduate School of Medicine, Kyoto University
Yoshida Konoe-cho, Sakyo-ku, Kyoto 606-8501, JAPAN
Phone: 81-75-753-9525; Fax: 81-75-753-9500
E-mail: haramari@kuhp.kyoto-u.ac.jp

Supplementary Fig. S1

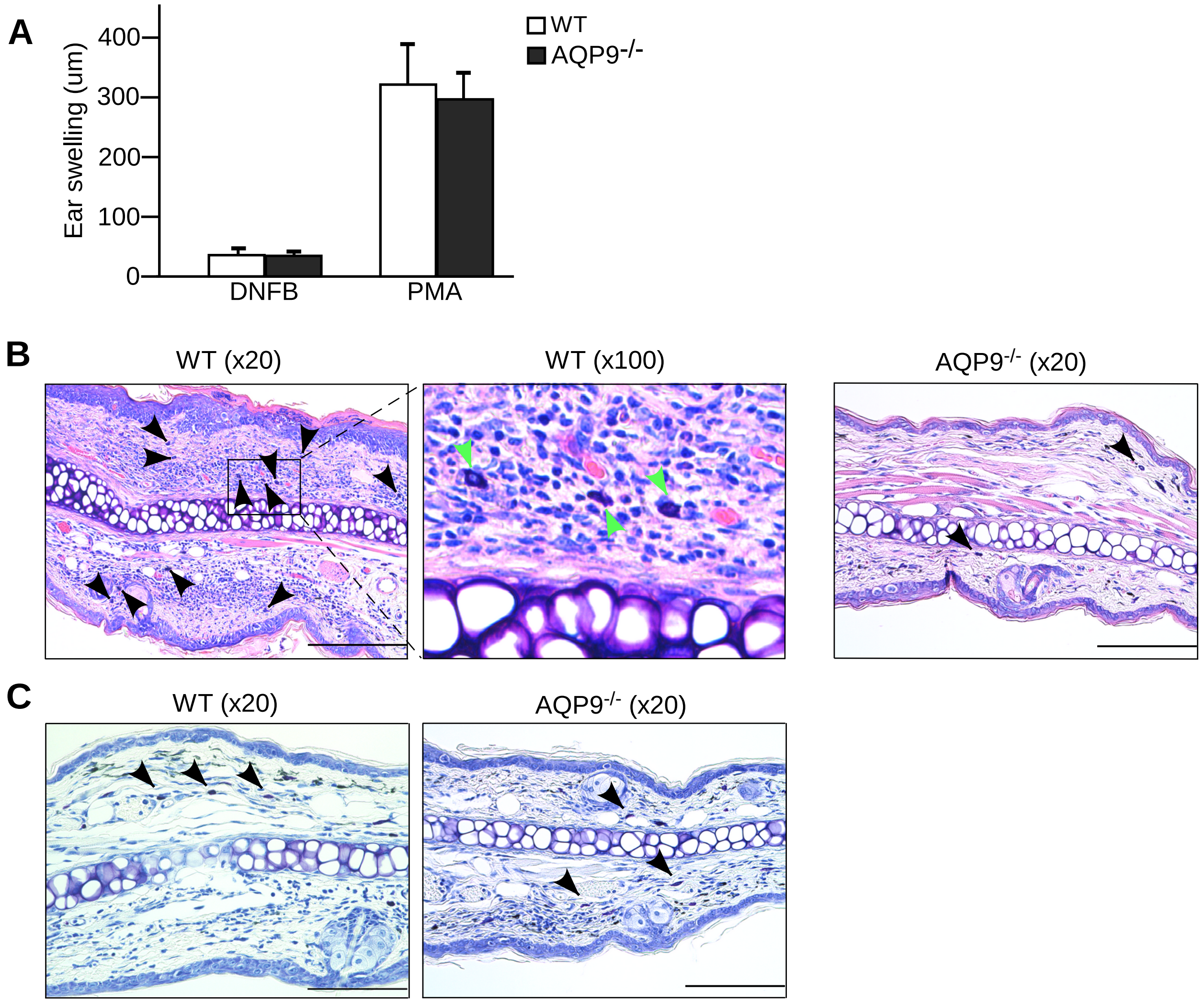

Supplementary Fig. S1. (A) Ear thickness in WT and AQP9$^{-/-}$ mice with single topical application of 0.5% dinitrofluorobenzene (DNFB) or 0.2 mg/ml phorbol myristate acetate (PMA) at 24 h as a model of irritant contact dermatitis. Both of ear swellings were comparable (n=3-4).

(B, C) Mice were sensitized with DNFB, and challenged 5 d later on the ear. The ears were collected at 24 h after challenge. A representative image of neutrophils infiltration (arrow head) with Giemsa staining (B), and mast cell infiltration (arrow head) with Toluidine blue staining (C) in the DNFB-challenged ear of WT (left) and AQP9$^{-/-}$ (right) mice. Bar, 100 μm.

Supplementary Fig. S2

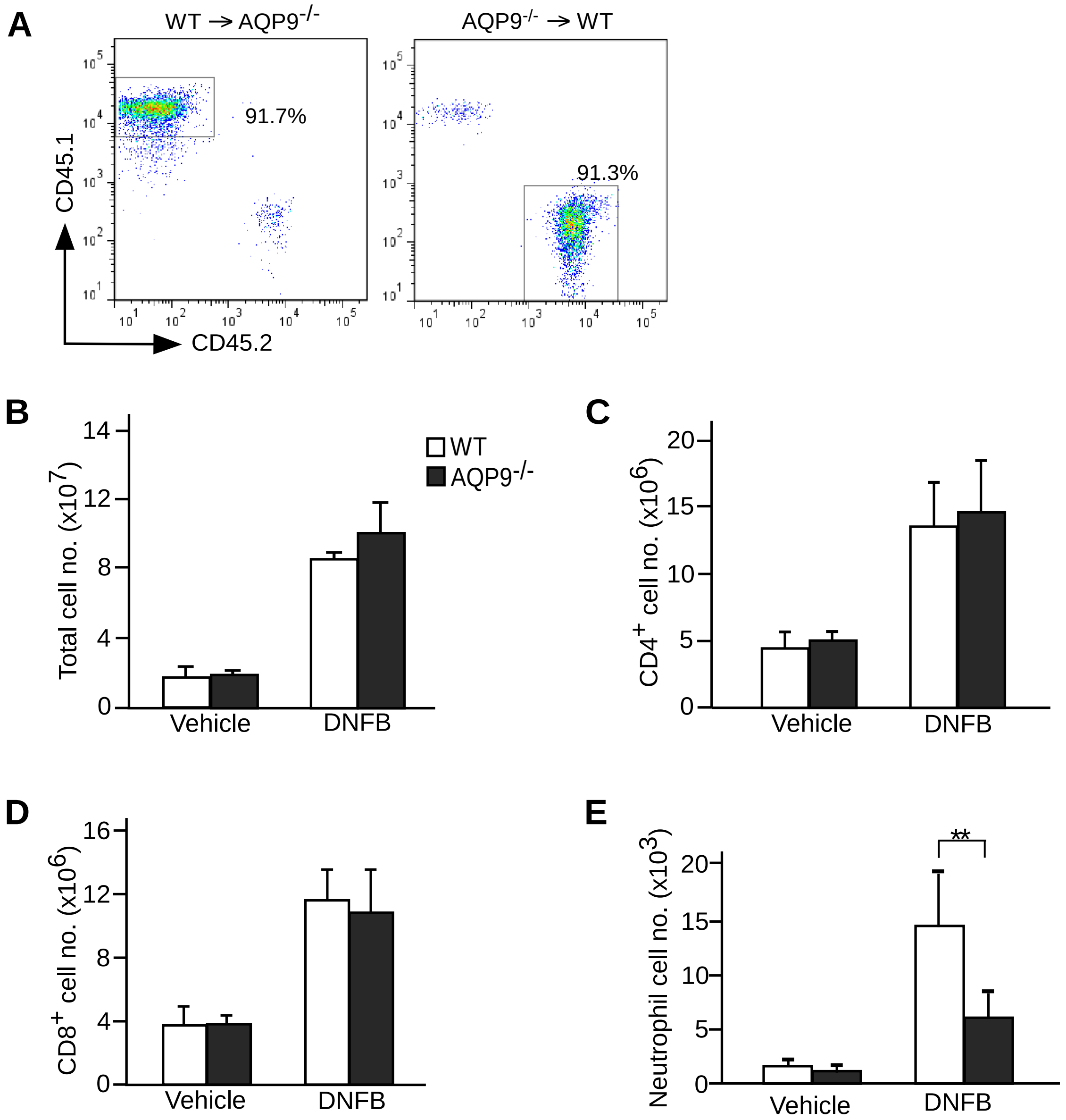

Supplementary Fig. S2. (A) Mice (WT or AQP9$^{-/-}$) were gamma-irradiated with two doses of 600rad, 3 hours apart. The irradiated mice received intravenous injection of bone marrow from WT or AQP9$^{-/-}$ mice. The chimerism was checked using C57BL/6 CD45.1 congenic mice. Two months after bone marrow transfer, blood cells were stained with CD45.1-pacific blue and CD45.2-APC, and analyzed by flow cytometry. More than 90% cells of the recipient cells were replaced by donor cells. (B-E) The compositions of skin dLNs cells after sensitization with 0.5% DNFB. Five days after sensitization, the total cell numbers (B), CD4^{+}cells (C), and CD8^{+} cells (D) were comparable in the skin dLNs between WT and AQP9$^{-/-}$ mice; while the neutrophil numbers (E) were increased in WT compare to AQP9$^{-/-}$ mice (n=4 per group; **, $p<0.01$).

Supplementary Fig. S3

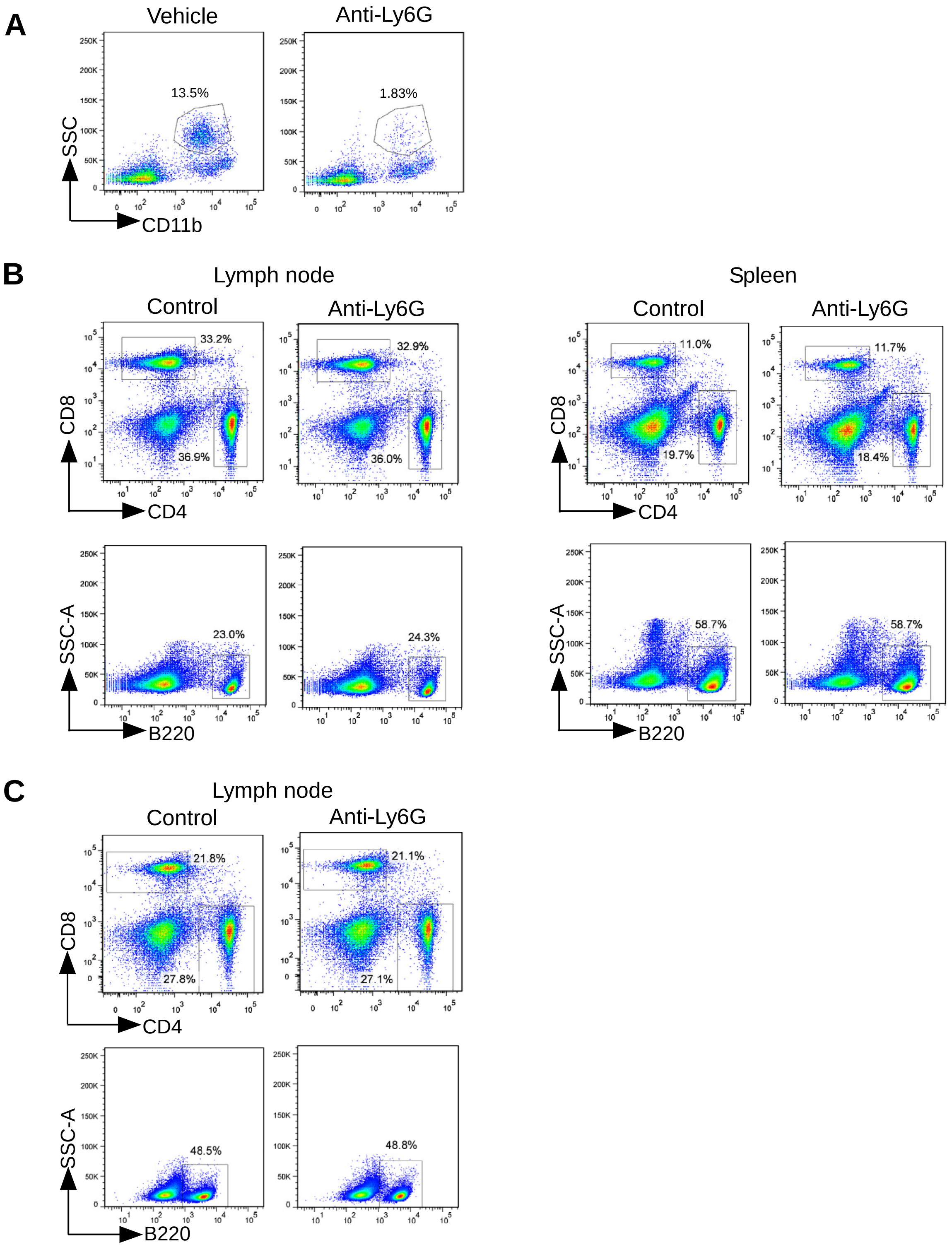

Supplementary Fig. S3. Effects of neutrophil depletion during CHS.
(A) Representative FACS analysis of SSC^{high} $CD11b^{+}$ neutrophils in the blood of mice injected with anti-Ly6G mAb at day 5. B-C. Representative FACS analysis of $CD4^{+}$, $CD8^{+}$, and $B220^{+}$ cells in spleen and/or skin dLNS of WT mice in the steady state (B) and after DNFB sensitization (C) with or without anti-Ly6G injection.

Supplementary Fig. S4

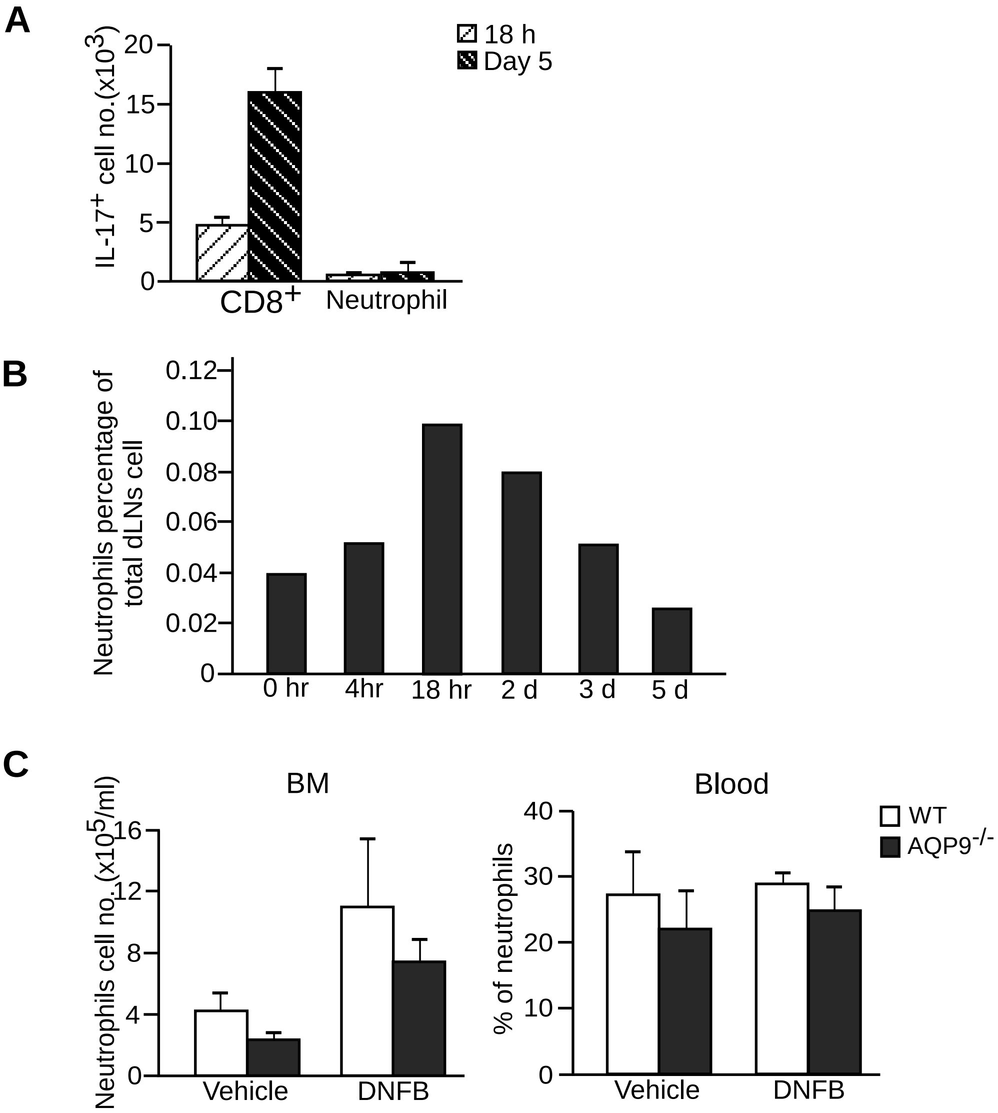

Supplementary Fig. S4. (A) Skin dLNs were collected at 18 h and 5 d after DNFB sensitization in WT mice for intracellular IL-17A staining by flow cytometry analysis.
LN cells were cultured for 2 h with PMA (50ng/ml) and ionomycin (1μM) in the presence of GolgiStop (n=3 per group). (B) 0.5% DNFB was applied to shaved abdomen skin of WT mice at indicated times, and the percentage of neutrophil ($Gr1^+$ $CD11b^+$) infiltration in skin dLNs was calculated by flow cytometry. (C) Bone Marrow (BM) and blood cells were collected at 18 h after DNFB application. Neutrophils ($Gr1^+$ $CD11b^+$) cell numbers in BM (left) and the ratio in blood (right) were comparable between WT and $AQP9^{-/-}$ mice as analyzed by flow cytometry (n=4 per group).

Supplementary Fig. S5

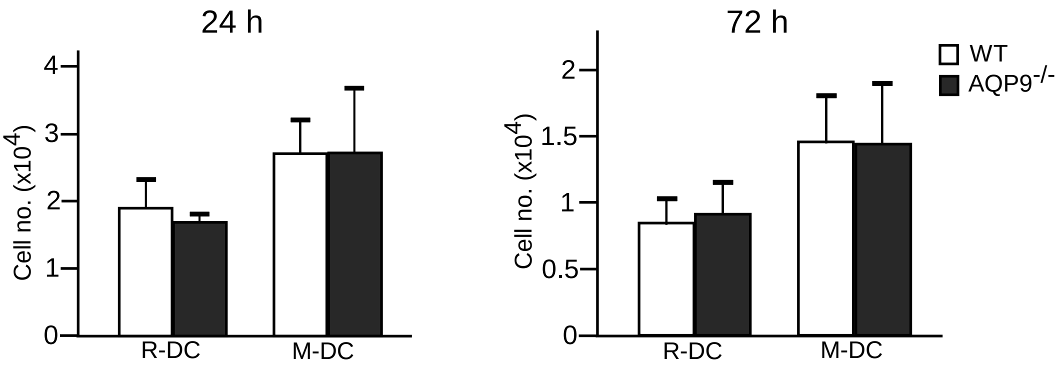

Supplementary Fig. S5. FITC-induced cutaneous dendritic cell (DC) migration assay. Mice were painted on the shaved abdomen with 200 μl of 1% FITC in 1:1 (v/v) acetone/dibutyl phalate mixture. The number of $CD11c^{+}$ MHC class II^{+} $FITC^{+}$ cutaneous DCs that migrated into the skin dLNs was enumerated by flow cytometry. The migrated cell numbers were comparable between WT and $AQP9^{-/-}$ mice, either at 24 h (left) and 72 h (right) after the application of FITC (n=4-5 per group). R-DC = resident DC; M-DC = migrated DC.

Supplementary Fig. S6

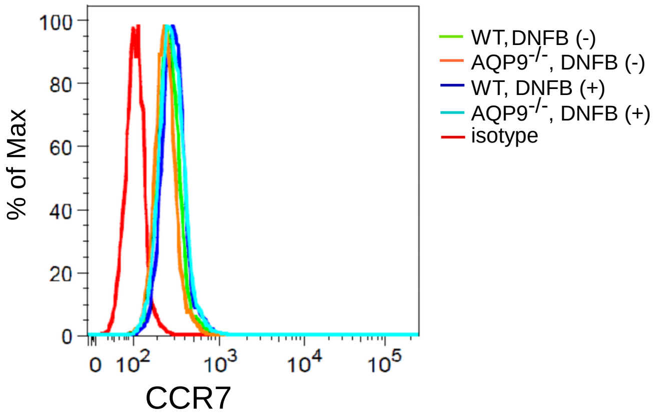

Supplementary Fig.S6. CCR7 expression on neutrophils from WT and $AQP9^{-/-}$ mice with or without DNFB sensitization. CCR7 expressions were similar between each group.
